# Effect of Preoperative Inflammatory Status and Comorbidities on Pain Resolution and Persistent Postsurgical Pain after Inguinal Hernia Repair

**DOI:** 10.1155/2016/5830347

**Published:** 2016-03-09

**Authors:** Dario Bugada, Patricia Lavand'homme, Andrea Luigi Ambrosoli, Gianluca Cappelleri, Gloria MR Saccani Jotti, Tiziana Meschi, Guido Fanelli, Massimo Allegri

**Affiliations:** ^1^Department of Surgical Sciences, University of Parma, Via Gramsci 14, 43126 Parma, Italy; ^2^SIMPAR Group (Study in Multidisciplinary Pain Research Group), Via Gramsci 14, 43126 Parma, Italy; ^3^2° Anestesia, Rianimazione e Terapia Antalgica, Azienda Ospedaliero-Universitaria di Parma, Via Gramsci 14, 43126 Parma, Italy; ^4^Department of Anesthesia and Perioperative Medicine, Catholic University of Louvain, St Luc Hospital, 10 Avenue Hippocrate, 1200 Brussels, Belgium; ^5^Day Surgery Unit, Azienda Ospedaliera Ospedale di Circolo e Fondazione Macchi, Polo Universitario, Viale Luigi Borri 57, 21100 Varese, Italy; ^6^Department of Anesthesia, Istituto Ortopedico G. Pini, Piazza Cardinale Andrea Ferrari 1, 20122 Milan, Italy; ^7^Department of Biomedical, Biotechnological & Translational Sciences (S.Bi.Bi.T), Faculty of Medicine, University of Parma, Via Gramsci 14, 43126 Parma, Italy; ^8^Department of Clinical and Experimental Medicine, University of Parma, Via Gramsci 14, 43126 Parma, Italy

## Abstract

Poor acute pain control and inflammation are important risk factors for Persistent Postsurgical Pain (PPSP). The aim of the study is to investigate, in the context of a prospective cohort of patients undergoing hernia repair, potential risk factors for PPSP. Data about BMI, anxious-depressive disorders, neutrophil-tolymphocyte ratio (NLR), proinflammatory medical comorbidities were collected. An analysis for correlation between comorbidities and PPSP was performed in those patients experiencing chronic pain at 3 months after surgery. Tramadol resulted less effective in pain at movement in patients with a proinflammatory status. Preoperative hypertension and NLR > 4 were correlated with PPSP intensity. Regional anesthesia was significantly protective on PPSP when associated with ketorolac. Patients with pain at 1 month were significantly more prone to develop PPSP at 3 months. NSAIDs or weak opioids are equally effective on acute pain and on PPSP development after IHR, but Ketorolac has better profile in patients with inflammatory background or undergoing regional anesthesia. Drug choice should be based on their potential side effects, patient's profile (comorbidities, preoperative inflammation, and hypertension), and type of anesthesia. Close monitoring is necessary to early detect pain conditions more prone to progress to a chronic syndrome.

## 1. Introduction

Persistent postsurgical pain (PPSP) influences patients' quality of life (even after minor surgical procedures) [1–3] and is currently regarded as an important outcome reflecting the quality of perioperative care provided to patients. A recent study just confirmed that moderate-to-severe PPSP is common after outpatient surgery (i.e., ambulatory and short-stay procedures) [4], and inguinal hernia repair (IHR) is a very common surgical procedure, often performed on an ambulatory base.

Among risk factors for PPSP development, the severity of acute postoperative pain is often mentioned, which implies that individuals prone to suffer intense postoperative pain may be the most vulnerable to PPSP [5, 6]. However, not every patient with severe postoperative pain will later develop PPSP. The use of pain trajectories, which offer a dynamic view of postoperative pain, allows unmasking abnormal pain resolution susceptible to lead towards PPSP [7, 8].

Postoperative pain involves an inflammatory reaction and its nonresolution might be an important determinant of both acute pain severity and pain persistence after surgery [9, 10]. Peripheral inflammation and chronic neural inflammation influence both excitatory and inhibitory signaling along pain pathways, thus having a major role in hyperalgesia and central sensitization [9, 10]. On the other hand, the systemic, enhanced proinflammatory status has to be regarded as an important determinant of vulnerability: conditions such as irritable bowel syndrome, migraine headache, fibromyalgia, Raynaud disease, and obesity are frequently found in the history of patients presenting with PPSP. These situations are in accordance with the hypothesis of an algesic proinflammatory priming necessary for the development of PPSP [11]: according to this theory, modifications of the proinflammatory/anti-inflammatory balance in some patients may favor the development of PPSP, but clear evidence is still lacking [12].

Furthermore, the relation between pain and blood pressure has been partially documented. Pain and cardiovascular modulatory pathways are overlapped and connected [13, 14], and correlations between resting blood pressure and pain sensitivity have been already documented in previous studies, with differences between acute and chronic pain patients [13, 15, 16]; moreover, a correlation between metabolic syndrome (hypertension being part of this) and chronic pain is well known [17]. However, no studies, to our knowledge, have investigated whether preexisting hypertension is correlated with PPSP.

Since physicians are still seeking tools to target patients at risk for PPSP, it is reasonable to investigate whether the above-mentioned preoperative conditions can help in identifying patients more prone to develop PPSP and whether analgesic strategies have different efficacy according to basal inflammation's level. We thus performed a longitudinal study in the context of a prospective cohort of patients receiving a specific type of surgery. The aim of this study was to assess any predictive value of preoperative BMI, hypertension, anxious depressive disorders, and proinflammatory status on PPSP (both* incidence* and* intensity*) and to verify any difference in the efficacy of perioperative analgesic treatment like ketorolac and tramadol/acetaminophen according to the patient's systemic proinflammatory status. Pain trajectories were used to evaluate the evolution of pain over the period of observation and investigate the effect of acute pain on pain resolution over three months.

## 2. Methods

We performed a longitudinal analysis on patients previously enrolled for a prospective, single blind, open-label clinical trial, registered on Clinicaltrials.gov (NCT01345162, Principal Investigator: Massimo Allegri) and EUDRACT (2009-011-856-23). Two hospitals enrolled all of the patients (IRCCS Policlinico S. Matteo, Pavia, Italy, and Ospedale di Circolo e Fondazione Macchi, Varese, Italy), from March 2010 to May 2012.

Methods and results of our randomized clinical trial can be retrieved on a previous paper published by our group [18]. In summary, adult patients scheduled for monolateral inguinal hernia repair with anterior approach (open, non-video-laparoscopic approach) and tension-free technique (mesh repair) received either general, spinal, or local anesthesia with field infiltration and were then randomized into two groups: (1) ketorolac 30 mg iv every eight hours for the first 24 hours after surgery and then ketorolac 10 mg per os every eight hours for three days after discharge or (2) tramadol 100 mg iv (50 mg if weighing less than 50 kg) every eight hours for the first 24 hours after surgery and tramadol 37.5 mg/paracetamol 325 mg association (Patrol®) per os every eight hours after discharge. Rescue analgesia was provided with paracetamol 1000 mg in both treatment groups.

For the aim of the current study, we used a combination of prospectively and retrospectively collected data.

Preoperative data included patient's age, gender, body mass index (BMI), patient's medical history including the presence of a chronic pain condition, inflammatory conditions (i.e., irritable bowel syndrome, migraine, and rheumatologic diseases), preoperative arterial hypertension, and anxious depressive disorders under treatment. All of these data were prospectively registered during preoperative anesthesiology consultation and were therefore available for all the patients included in the study; data were recorded on a specific form used by all anesthesiologist as part of anesthesia chart, encompassing all medical history of the patient (including allergies, previous surgeries, comorbidities, and demographic and anthropometric data). We registered all those conditions only once a previous diagnosis was already defined by other clinicians (not just trusting in patients' report).

Since all patients scheduled for hernia repair in our institutions routinely receive preoperative blood analyses, the preoperative neutrophils-to-lymphocytes ratio (NLR) was retrospectively retrieved from clinical charts and considered as a marker of systemic inflammation.

Pain evaluations were performed by blinded observer (details about blinding are available in our previous paper [18]). All the patients were evaluated at the end of surgery at PACU arrival (T0) and then at 6, 12, and 24 hours after surgery. Pain intensity was assessed using 11-point numeric rating scale (NRS: 0 “no pain” to 10 “worst possible pain”). Pain was measured at rest (NRS) and when moving (mNRS), that is, deep inspiration and coughing (dynamic pain). The patients were sent home with a “pain diary” to be completed from day one to day five and were asked to record details of pain. On day five after surgery, all the patients came back for a surgical examination. Pain scores at rest and during movement were summarized, together with the need for rescue analgesics.

At one month and three months from surgery, a phone interview was performed to ask the patients if they were complaining from pain in the area of surgery (PPSP, according to IASP definition [19]).

A visit to the pain center was scheduled for PPSP patients for a detailed evaluation and for proper treatment. Pain intensities at rest and with mobilization were assessed, and a surgical evaluation was carried out to eliminate any surgical complication as the main cause of pain persistence (wound healing problem, infection, prosthesis' reaction, and hernia recurrence).

## 3. Statistical Analysis

For analysis of risk factors, we have considered as* affected by PPSP* only patients with an NRS score ≥ 3/10 at rest and/or during movement, that is, patients who reported at least moderate PPSP at one or three months after inguinal hernia repair. Then, the patients (with and without PPSP) were separated into four groups according to the postoperative analgesic treatment, that is, tramadol or ketorolac.

The* preoperative proinflammatory status* was defined as either the presence or combination of BMI ≥ 30 kg/m^2^, NLR ≥ 4, or a proinflammatory medical condition (rheumatologic disease, bowel disease, Crohn's disease, and migraine headache).

Data were summarized within groups as mean and standard deviation (SD) for continuous variables and counts and % for categorical variables. According to a Kolmogorov-Smirnov normality test, parametric data between the groups were compared by unpaired Student's *t*-test and nonparametric data by Mann-Whitney rank sum test or Kruskal-Wallis one-way analysis of variance on ranks with Dunn's Method for pairwise multiple comparison; a *p* value <0.05 was considered to be significant (SigmaStat 3.5, Systat Software GmbH, Erkrath, Germany). Categorical data were compared using Chi-square test and Fisher exact test using a two-tailed probability. Spearman rank order correlations were also used.

## 4. Results

Two hundred patients were enrolled in the clinical study. Two patients in group K and four patients in group T were excluded from the final analysis due to protocol violations (they did not accept the prescribed therapy or stopped it before the third day after surgery). Ninety-eight patients in group K and 96 in group T were included in the final analysis. Preoperative data were available for all the patients; we were able to retrieve NLR values in 80 patients of group K (81.6%) and 79 of group T (82.3%), with a total of 159 patients (82%) whose NLR values were available for this study.

Eighteen patients (9.3%) reported chronic pain at 3 months, 12 patients (12.2%) in group K and 6 patients (6.3%) in group T (Table 1).

**Figure 1:**
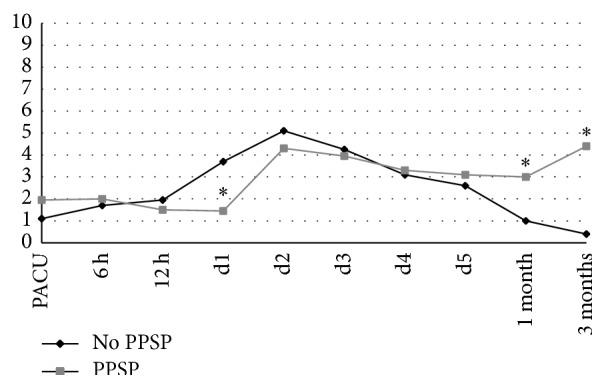
Pain trajectories of patients with and without persistent postsurgical pain (PPSP) from day 0 until 3 months after surgery.

We first evaluated evolution of pain over the time of observation. The pain trajectories for dynamic pain of patients from day 0 (PACU) until three months after surgery, with and without PPSP, are presented in Figure 1. From our data analysis, it seems that patients with PPSP (*n* = 18) had lower pain scores at day one (24 h after surgery) than patients who did not present with PPSP. At one month after surgery, patients who had PPSP already demonstrated higher pain intensity than patients without PPSP. Accordingly, the presence of PPSP at three months was statistically correlated with the presence of pain at one month after surgery (*r* = 0.408; *p* < 0.001).

Both postoperative treatments, ketorolac and tramadol, were effective in controlling acute pain. Data also seem to show that spinal anesthesia and local infiltration provide some protective effect against the development of persistent pain in patients receiving ketorolac (Table 1).

No positive correlation with PPSP* incidence* was found for the following factors, that is, BMI, hypertension, anxiety depressive disorders, and preoperative NLR value. Preoperative NLR value was 2.3 ± 0.9 (95% CI: 2.2) in patients without PPSP at 3 months versus 1.9 ± 0.3 (95% CI: 0.2) in patients with PPSP. Interestingly, when analyzing PPSP* intensity*, a positive correlation was found with the preoperative NLR value (*r* = 0.282; *p* = 0.02) as well as the presence of preoperative hypertension (*r* = 0.237; *p* = 0.0008).

### 4.1. Patients Stratification according to the Presence of a Preoperative Inflammatory Status

**Figure 2:**
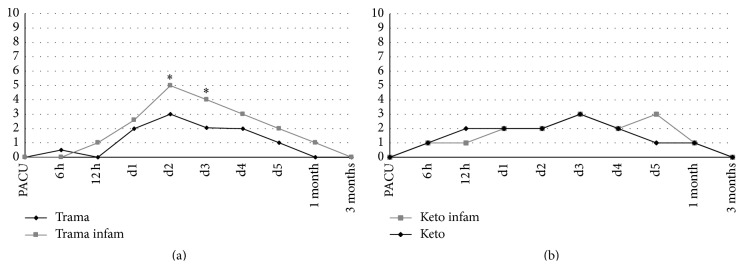
Pain trajectories at movement in patients with or without inflammatory background according to treatment group. (a) Ketorolac. (b) Tramadol. ^*∗*^ *p* < 0.05 between groups at day 2 and 3.

On the postoperative pain trajectories, patients with an established preoperative proinflammatory condition who were treated with tramadol had less effective pain control during mobilization and displayed significantly higher postoperative pain scores at day two and day three after surgery than patients without preoperative proinflammatory status (Figure 2(a)). No significant difference was found in group K patients (Figure 2(b)).

Regarding severe postoperative pain (expressed as NRS > 6/10 in the first 24 hours—Table 1), we retrieved no statistically significant difference between patients with proinflammatory background or not (7% versus 9%). As well, no significant difference was retrieved in PPSP incidence comparing patients with proinflammatory background or not, neither at 1 month (11% versus 13%, *p* = 0.086) nor at 3 months (4% versus 10%, *p* = 0.480). Finally, considering only patients with a proinflammatory status, no difference in PPSP incidence was noted according to ketorolac or tramadol use, neither at 1 month (0% versus 19%, *p* > 0.05) nor at 3 months (0% versus 6%, *p* > 0.05).

## 5. Discussion

Major interest exists towards PPSP, since it may be considered as an invalidating condition with severe impact on patients' quality of life [1–3, 20], as well as high costs for Health Systems even after minor procedures and procedures performed on a short-stay basis [4]. Ideally, patients at risk for persistent pain should be identified during the preoperative phase with the aim of getting tailored perioperative treatment [4].

The early postoperative period deserves also the attention as the severity of acute pain; that is, the failure of postoperative pain management seems to be the most striking risk factor for PPSP [5, 6, 21]. As demonstrated by the pain trajectories, the control of pain in the weeks following the surgical procedure is also important. We found that patients with subacute pain at one month were more prone to develop PPSP at three months, thereby indicating pain at one month as a predictive factor for the development of persistent pain even when dealing with minor surgical procedures associated with mild-to-moderate pain scores. It is noteworthy that patients suffering PPSP have lower pain scores at day 1 after surgery; this may result controversial, since most publications suggest that higher pain scores in the early postoperative period predict PPSP development. However, the question is, in our opinion, what “*early*” means; this time span should be better defined, since our data conflicts with previous studies when considering NRS values at day 1, but they do not when dealing with pain during the following days (pain is not significantly different between groups by the second day). We cannot explain such difference in the PPSP versus no PPSP patients at day 1, but what is more important (and innovative), in our opinion, is that the* dynamic* progression of pain (given by pain trajectories) predicts chronicization, and not the static measurement at a prespecified time point (such as day 1): patients in PPSP group (despite lower pain scores at day 1) display a tendency to reduced pain* resolution*, and pain trajectories tend to invert along time in the two groups. In other words, it is not pain at day 1 but the “nonresolution” of pain that leads to chronicization, and stratification basing on a single (static) evaluation may be misleading in anticipating PPSP development.

It is noteworthy that the “nonresolution” leads to significant differences in NRS already at 1 month: that time point is when anesthesiologists or surgeons usually lose patients to follow-up, but also when subacute pain (anticipating longer pain persistence) may be detected. Our data seems to strongly support the concept of dynamic evaluation of pain and, of course, the creation of PPSP-dedicated, dynamic pain facilities, in order to keep the pace with each patient's pain experience and detect those at risk of chronicization, even after minor surgical procedures.

Other risk factors for PPSP are currently a matter of debate. Since inflammation is one of the main determinants of peripheral/central sensitization and hyperalgesia (which are believed to be the main mechanisms involved in PPSP) [9], inflammatory processes are interesting targets to be investigated for early risk stratification/effective treatment of patients.

Our study did not demonstrate any relationship between basal inflammatory status and pain, neither acute nor chronic pain, with the only exception of preoperative NLR, which is correlated with higher PPSP* intensity*. Still, we cannot rule out the specific mechanism involved in this susceptibility. NLR may be directly influenced by cytokines productions [22] and was found to be an indicator of systemic inflammation. NLR displayed important value for outcome's prediction in cancer patients [23], as well as in cardiac diseases [24, 25]. A comprehensive theory proposed an algesic proinflammatory priming necessary for the development of PPSP, and chronic inflammatory conditions are a common finding in patients with PPSP [11]. Thus, we can postulate that higher NLR identifies patients with enhanced presurgical systemic inflammation, as well as being probably more exposed to the nonresolution of pain due to this altered inflammatory balance. Further, high NLR may also reflect an altered inflammatory balance within the central nervous system, in which inflammatory signaling is a major mechanism of hyperalgesia [9, 10]. However, this is, to our knowledge, the first study documenting this correlation, which probably deserves to be investigated in larger samples, or in patients with higher degrees of basal inflammation, or in the context of more invasive surgeries associated with increased systemic inflammatory response.

Furthermore, preoperative patients' stratification according to basal inflammation might be useful to predict analgesics' effectiveness and thus protect from PPSP: we observed better analgesia in patients with inflammatory background treated with ketorolac instead of tramadol, pointing out basal inflammation as a prognostic factor for proper drug choice (to identify patients more likely to be treated with NSAIDs). Inflammation is a base mechanism for transduction and transmission of pain stimuli, both at peripheral and at central site [9]; it is straightforward to suppose that the higher the inflammatory component, the bigger the advantage in using NSAIDs instead of tramadol (which does not display any effect on inflammation). It is noteworthy that ketorolac is mainly active on COX1 rather than on COX2, while central inflammation (estimated to contribute to pain/analgesia with an effect size of nearly 40% [26]) is mainly COX2 mediated [9]: this lays the doubt about the efficacy of a higher COX-2-selective NSAID (i.e., etoricoxib, diclofenac) on pain, especially in patients with higher preoperative NLR; further studies should be addressed to this issue.

The association between local/spinal anesthesia and ketorolac shows to be protective against PPSP. Still, the protective effect may be enhanced by using more COX2-selective NSAIDs (such as diclofenac) because of their higher specificity towards mechanisms involved in central hyperalgesia and sensitization [9]. However, our results endorse the concept of regional techniques as useful in protective analgesia and may justify, once confirmed by further studies, the choice of NSAIDS instead of other drugs when systemic and regional analgesia are combined for hernia repair [12].

Except for NLR, the only other independent risk factor for PPSP intensity was the preoperative arterial hypertension. No other correlations were retrieved for single comorbidities usually identified as more prevalent in the chronic pain-affected population (higher BMI [27], anxious depressive disorders [28], irritable bowel syndrome, Crohn's disease, migraines, and rheumatologic diseases [12]).

The relationship between hypertension and chronic pain is still not clear, but pain and cardiovascular modulatory pathways are overlapped and connected [13, 29]. It is assumed that low resting BP is associated with greater pain sensitivity: such an association was observed in animal and human models of acute pain [15]. Otherwise, some studies on chronic pain patients have reported a positive correlation between blood pressure and pain sensitivity in chronic pain conditions [13, 30], as well as an increased prevalence of hypertension in chronic pain population [16], suggesting chronic pain as a risk factor for hypertension [31]. Our study further argues in favor of a high blood pressure-pain intensity association; additionally, to our knowledge, this is the first evidence that hypertensive patients undergoing surgery might become more prone to suffer higher levels of PPSP.

We cannot certainly rule out the mechanisms involved in this process only by basing on the current data, but we speculate that the hypertension-PPSP relationship observed in our population may reflect a pathologic, maladaptive mechanism in the common adrenergic pathway (pain and blood pressure), leading to hypertension and central sensitization (and persistent pain). Interactions in blood pressure/pain pathways are believed to reflect a homeostatic feedback based on baroreceptors, which activate descending pain inhibition once stimulated by elevated blood pressure [15], resulting in reduced pain sensitivity and thus facilitating the return to a normal homeostatic state. Research suggests that this blood pressure/pain regulatory relationship may be substantially altered in chronic pain conditions [31]; this feedback mechanism may be altered in some patients, therefore not resulting in pain inhibition in the postoperative setting but in an opposite effect (hyperalgesia/absence of antinociception), which may be the base for the nonresolution of the ongoing pain state.

We found that ketorolac had better analgesic profile in patients with an inflammatory background and in patients undergoing regional anesthesia. Hypertension and NLR were, for the first time, identified as risk factors for PPSP intensity. Larger studies with accurate patient selection are required to confirm these data; however, preoperative stratification of patients at risk and targeted therapies according to basal inflammation should be considered to improve postoperative pain relief and protection from PPSP. Finally, independently of risk factors, close monitoring is necessary to identify subacute pain conditions, which are more prone to progress to a chronic, invalidating situation.

This study has several limitations. First, NLR data were retrospectively collected; however, we retrieved data of more than 80% of patients for analysis of correlations, partially overcoming this issue. Second, the cut-off value for NLR was chosen according to the majority of studies on cancer patients; although no defined cut-off values have been established so far, even for cancer outcomes, this is the best data available in literature. Since hernia patients are on a benign disease (not associated with the enhanced, malignancy-associated inflammatory response), this may influence our results, and different cut-off should be established for this kind of patients: further studies are needed to clarify this issue. Third, the actual sample size for this study is limited to patients experiencing PPSP, that is, a reduced part of the larger trial population. This may limit to some extent the strength of our findings and the ability to make definitive conclusions; however, this is, to our knowledge, one of the biggest populations analyzed for correlations between preoperative comorbidity and persistent pain, with homogeneous surgical procedure and postoperative treatment. Fourth, the number of patients with PPSP considered for analysis may theoretically be higher: actually, we considered only patients with NRS ≥ 3/10 at rest and/or during movement (i.e., patients who reported at least moderate PPSP and a “clinically significant” persistence of pain). Once* all* patients with pain at 1 or 3 months are included (whether clinically impactful or not), population gets wider: indeed, this larger population may provide more significance to our results, but it might also establish less clinically meaningful correlations. Finally, the PPSP group in the ketorolac group is approximately 10 years younger than all other groups (Table 1): increasing age is associated with decrease in pain perception and might be of significance in this cohort.

## Figures and Tables

**Table 1:** Characteristics of patients with and without persistent postsurgical pain (PPSP) at 3 months after surgery according to postoperative group of treatment.

	Group ketorolac		Group tramadol	
	No PPSP	PPSP	No PPSP	PPSP
	(*n* = 86)	(*n* = 12)	(*n* = 90)	(*n* = 6)
Age (years)	58 ± 14	49 ± 14	56 ±15	58 ± 11
BMI (kg/m^2^)	25 ± 2.4	25 ± 2.8	25 ± 3.0	26 ± 3.0
Preoperative NLR	2.3 ± 0.9	1.85 ± 0.2	2.3 ± 0.9	1.87 ± 0.5
Proinflammatory condition (*n*)^†^	8 [8%]	0 [0%]	9 [10%]	1 [17%]
Hypertension (*n*)	20 [23%]	5 [42%]	21 [23%]	2 [33%]
Anxiety depression (*n* - %)	7 [8%]	1 [8%]	8 [9%]	1 [17%]
Postoperative NRS ≥ 6/10 at 24 h (*n*)	8 [9%]	1 [13%]	7 [8%]	0 [0%]
Type of anesthesia (*n*)				
General	22 [26%]	2 [17%]	20 [22%]	0 [0%]
Spinal	40 [47%]	1 [8%]^*∗*^	46 [51%]	2 [33%]
Local infiltration	24 [28%]	9 [75%]^*∗∗*^	24 [27%]	4 [67%]

^*∗*^ *p* = 0.01 between patients with PPSP and patients with no PPSP in ketorolac group of treatment; ^*∗∗*^ *p* = 0.02 between patients with PPSP and patients with no PPSP in ketorolac group of treatment. There were no other statistically significant differences among the groups presented in the table. ^†^Proinflammatory condition was defined as follows: either the presence or combination of BMI ≥ 30 kg/m^2^, NLR ≥ 4, or the existence of a proinflammatory medical condition (rheumatologic disease, bowel disease, Crohn's disease, and migraine headache).
